# A Review of Recent Advances in the Management of Alzheimer’s Disease

**DOI:** 10.7759/cureus.58416

**Published:** 2024-04-16

**Authors:** Skylynn Thangwaritorn, Christopher Lee, Elena Metchikoff, Vidushi Razdan, Suliman Ghafary, Dominic Rivera, Alvaro Pinto, Sudhakar Pemminati

**Affiliations:** 1 Department of Biomedical Education, California Health Sciences University College of Osteopathic Medicine, Clovis, USA

**Keywords:** beta-amyloid peptide, orexin, amyloid plaques, cholinesterase inhibitors, dementia

## Abstract

Alzheimer’s disease (AD) is the most common neurodegenerative condition and a form of dementia encountered in medical practice. Despite many proposed and attempted treatments, this disease remains a major puzzle in the public health systems worldwide. The initial part of this article provides an overview and illustration of the primary mechanisms responsible for neuronal damage in AD. Subsequently, it offers a critical evaluation of the most noteworthy studies on pharmacological therapy for AD and outlines recent advancements and novel approaches to managing this condition. Main properties, categorization, Food and Drug Administration (FDA) status, mechanisms of action, benefits, and common side effects of the classical and the most recently proposed pharmacological treatments for AD are described. The conventional pharmacological agents revised comprise cholinesterase inhibitors, monoclonal antibodies, and other therapies, such as memantine, valproic acid, and rosiglitazone. The innovative reviewed pharmacological agents comprise the monoclonal antibodies: donanemab, gantenerumab, solanezumab, bapineuzumab, crenezumab, and semorinemab. Nutritional supplements such as alpha-tocopherol (vitamin E) and caprylidene are also revised. Tau and amyloid-targeting treatments include methylthioninium moiety (MT), leuco-methylthioninium bis (LMTM), an oxidized form of MT, and tramiprosate, which inhibits the beta-amyloid (Aβ) monomer aggregation into toxic oligomers.

Antidiabetic and anti-neuroinflammation drugs recently proposed for AD treatment are discussed. The antidiabetic drugs include NE3107, an anti-inflammatory and insulin sensitizer, and the diabetes mainstream drug metformin. The anti-neuroinflammatory AD therapies include the use of sodium oligomannate (GV-971), infusions with intravenous immunoglobulin aiming to decrease plasma levels of the constituents of Aβ plaques, and masitinib, a tyrosine kinase inhibitor that impacts mast and microglia cells. Additional anti-inflammatory agents being currently tested in phase-2 clinical trials, such as atomoxetine (selective norepinephrine reuptake inhibitor), losartan (angiotensin 2 receptor agonist), genistein (anti-inflammatory isoflavone neuroprotective agent), trans-resveratrol (polyphenol antioxidant plant estrogen), and benfotiamine (synthetic thiamine precursor), were reviewed. Lastly, drugs targeting Alzheimer's-associated symptoms, such as brexpiprazole (serotonin dopamine activity modulator) and suvorexant (orexin receptor antagonist), respectively, used for agitation and insomnia in AD patients, are reviewed. As experimental investigations and clinical research progress, there is a possibility that a combination of newly tested medications and traditional ones may emerge as a promising treatment option for AD in the future.

## Introduction and background

**Figure 1:**
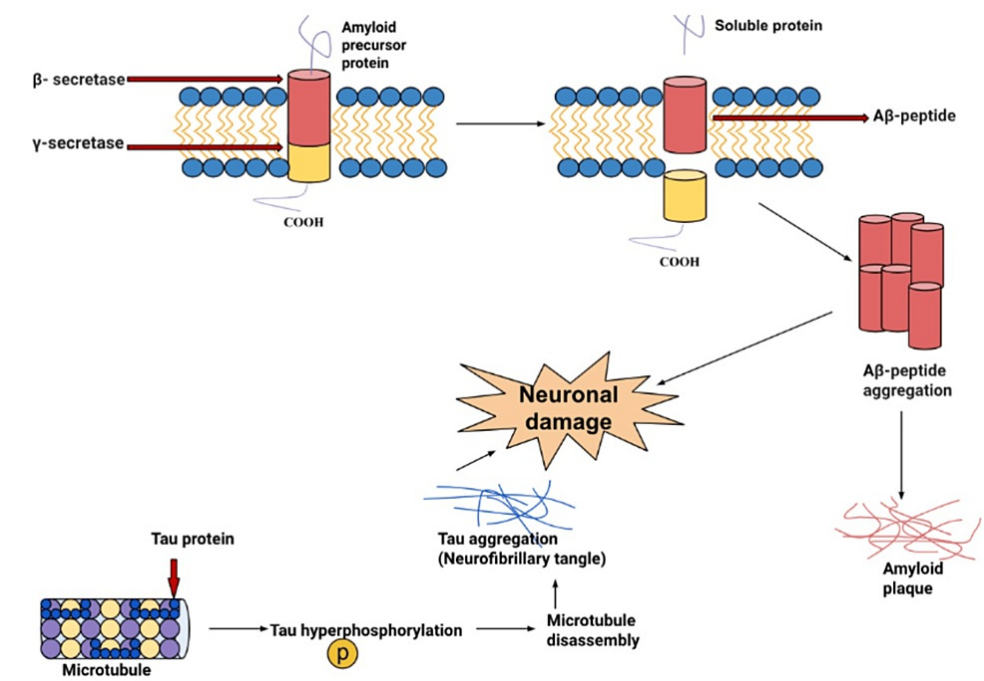
Mechanisms of Alzheimer’s disease neuronal damage Image credits: Christopher Lee, one of the authors of this study.

Alzheimer’s disease (AD) is the most common neurodegenerative disorder among adults. The development of this condition is indicated due to a progression of the deterioration of cognition, behavior, and functionality [1]. Compared to other disorders, the most prominent histopathological feature of AD is the accumulation of abnormally folded proteins in the brain as illustrated in Figure 1. The high concentration of the abnormal proteins results in the formation of intracellular neurofibrillary tangles and extracellular amyloid plaques [2]. The current theories that revolve around the pathophysiology of AD are based on genetic and neuropathological findings. They indicate that there is abnormal processing of the central molecular events of two proteins: amyloid precursor protein (APP) and tau. The existing neuropathological, genetic, and molecular biological evidence supports the neurobiological theory that there is an impact on the cascade of events that maintain the central roles for age-related changes in the metabolism of APP and tau protein. As a result, there is an accumulation of aggregates of beta-amyloid (Aβ) fibrils and neurofibrillary tangles in the brain. These aggregates affect neuronal and synapse function and integrity in selective brain regions leading to cognitive impairment [3]. AD can be identified genetically via causative agents such as APP, Presenilin 1, and Presenilin 2 genes (PS1/PS2). These causative genes encode amyloid beta peptide and lead to its accumulation in the brain. AD’s risk factors include low educational attainment, midlife hypertension, midlife obesity, hearing loss, late-life depression, diabetes, physical inactivity, smoking, and social isolation [4]. The strongest genetic risk factor for sporadic AD is apolipoprotein E (APOE) [5].

The limited availability of acetylcholine increases the chance of cognitive decline. This was targeted in the early treatments of AD by cholinesterase inhibitors. Overall, cholinesterase inhibitors are the most extensively evaluated drugs for AD and are a target for future treatments as well. Later came the neuroprotective agent memantine, which is an N-methyl-D-aspartate (NMDA) antagonist that is typically taken concurrently with a cholinesterase inhibitor [6]. Many novel and future treatments focus on monoclonal antibodies and various neuroprotective treatments.

**Figure 2:**
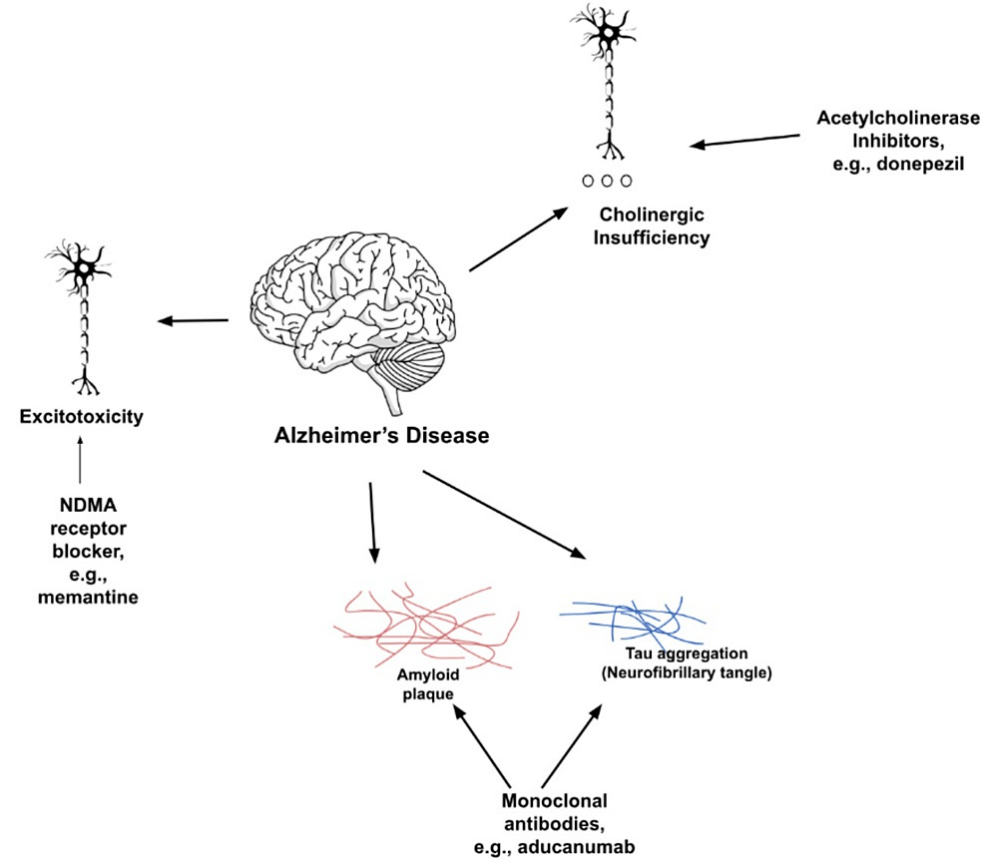
Mechanisms of current Alzheimer’s disease medications Image credits: Christopher Lee, one of the authors of this study.

Within this systematic review, we aim to create an overview of the pathophysiological processes of AD. Furthermore, we compare current drugs on the market to highlight the benefits and risks of the individual choice of certain drugs. Finally, we outline potential future drugs that may impact the landscape of efficacious treatments for AD beyond our current drug availabilities. Expanding our options for AD treatments allows more advanced treatments that may one day grant individuals a return to their baseline state before Alzheimer's. Growing research has focused on improved monoclonal antibodies with greater benefit to the patient with the least number of adverse effects possible. Monoclonal antibody-mediated removal of plaques is illustrated in Figure 2. Drugs that target tau and amyloid buildup, anti-inflammatory and neuroprotective agents, antidiabetic agents, antihypertensives, enzyme inhibitors, antivirals, and dopamine antagonists are also being developed. Correspondingly, this review seeks to provide future researchers with a better understanding of past and progressive treatments to springboard ideas for additional targets of action alleviating AD brain processes.

## Review

Methods

**Figure 3:**
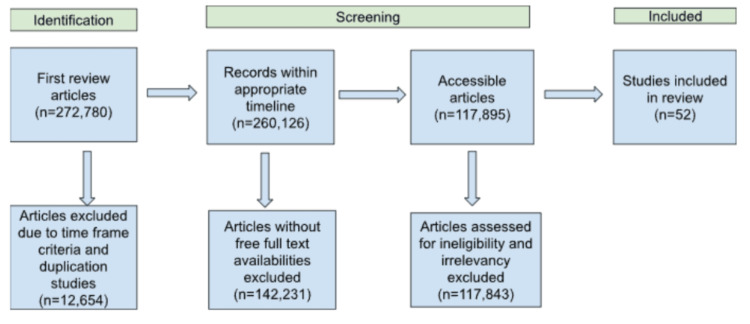
Flow chart of inclusion and exclusion criteria Image credits: Skylynn Thangwaritorn, one of the authors of this study.

PubMed/MEDLINE (Medical Retrieval Analysis and Retrieval System Online), Scopus, Web of Science, Elsevier, and Google Scholar databases were accessed to retrieve relevant literature on March 15, 2023. We have utilized the following keywords: "Alzheimer's disease," OR “Alzheimer's disease pharmacotherapy,” OR “Novel Alzheimer’s treatments,” OR “monoclonal antibodies for Alzheimer’s disease,” OR “Alzheimer’s treatments targeting Tau,” OR “Alzheimer’s Anti-inflammatory drugs,” OR “Alzheimer’s supplements.” The search was limited to phase three clinical trials and meta-analyses published in peer-reviewed journals for medications currently available. Phase one and two clinical trials were used for developing medications to indicate effectiveness in a smaller population. From selected studies, relevant information such as mechanism of action, benefits, and side effects were reviewed and summarized to provide a comprehensive overview of pharmacological treatments for AD, highlighting both established and emerging therapies. Duplication studies and articles that were not available in English were excluded. Additionally, for upcoming treatments, only trials from 2015 until 2023 were obtained. From there, a manual selection for appropriate drugs and drug categories was done to ensure the inclusion of any additional publications or trials that might have been missed by the electronic search and the exclusion of any articles ineligible or irrelevant to the systematic review (Figure 3).

The following sections are grouped as established current AD treatments and ongoing advancements in AD. Current treatments are divided into subsets of cholinesterase inhibitors, monoclonal antibodies, other treatments that did not fit into the previous two drug classes, and nutritional supplements. The ongoing advancement section was further categorized into monoclonal antibodies, tau and amyloid-targeting treatments, anti-neuroinflammation, antidiabetic, and miscellaneous drugs, phase 2 trials of anti-inflammatory agents, and AD-associated symptom treatments.

Current treatments

Acetylcholinesterase Inhibitors

Acetylcholinesterase inhibitors (AChEIs) increase the availability of acetylcholine in neuromuscular junction to reverse impaired cholinergic pathways seen in AD. As AChEIs increase acetylcholine in the entire body, their adverse effects closely resemble parasympathetic nervous system (PNS) overstimulation such as bradycardia, diarrhea, hypotension, and urinary incontinence. Because of these adverse effects, AChEIs are contraindicated in patients with pre-existing hypotension and cardiac conduction disease as increased PNS stimulation increases the risk of syncope [7]. Given the fragility of AD patients, syncope can increase the risk of fractures and concussions. However, there was no association between ChEIs and increased risk of urinary incontinence [8]. Additionally, AChEI uses were associated with an increased risk of angina, myocardial infarction, and stroke [9]. Therefore, close monitoring is required.

Donepezil

Donepezil is an oral medication that acts as a reversible cholinesterase inhibitor with a high affinity for acetylcholinesterase (AChE). A meta-analysis completed by Zhang et al. compared about 2847 eligible patients with mild cognitive impairment across randomized (RCT) and non-randomized concurrent clinical trials (CCT). Efficacy was determined based on Mini-Mental Status Exam (MMSE), Alzheimer’s Disease Assessment Scale-Cognitive Subscale (ADAS-cog), and Montreal Cognitive Assessment (MoCA). The study determined that compared to the control group, patients who received donepezil showed statistically significant improvement across MMSE (standardized mean difference [SMD] 0.85, 95% CI: 0.40-1.31) and MoCA (SMD: 1.88, 95% CI: 0.32-3.45) [10]. Additionally, patients taking donepezil reported statistically significant adverse effects such as nausea, vomiting, diarrhea, fatigue, headache, and dizziness.

Rivastigmine

Rivastigmine is a commonly used oral medication for AD, which acts as a pseudo-irreversible AChEI, targeting both AChE and butyrylcholinesterase. Karaman et al. argued that rivastigmine is more effective due to its multiple targets [11]. With increasing amounts of AChE in the human brain, the less available acetylcholine increases the chances of AD. Hansen et al. compared rivastigmine with donepezil, highlighting that the patients who received rivastigmine had a statistically significant cognitive function as indicated by the reduction of Alzheimer's Disease Cooperative Study/Activities of Daily Living Inventory (ADCS-ADL) score (−14.9 vs. −12.8, respectively; P < 0.05) [12]. Additionally, in a 52-week trial, there was an improvement in cognitive functions and global clinical function. Rivastigmine does not require liver function monitoring as it does not have hepatotoxicity as an adverse effect [13]. Administration of acetylcholinesterase inhibitors have typically only been available as oral formulations, which account for the adverse effects that include nausea and vomiting. Han et al. provided information about the benefits of switching to the rivastigmine patch during treatment to reduce the first-pass effects, which should reduce the side effects of nausea and vomiting. The study concluded that patients who switched from the oral to the patch responded well and that 80% of patients who were previously not responding well to the oral formulation showed some improvement but did not show any deterioration in global functioning. However, it should be noted that the data concluded that it was not statistically significant. Additionally, the patch form of rivastigmine can cause some mild skin irritation and might not be suitable for sensitive skin. In patients who cannot tolerate oral administration of rivastigmine, an immediate switch to the skin patch should be considered as an alternative route of administration [14].

Galantamine

Galantamine is an oral medication used for the treatment of mild to moderately severe Alzheimer's disease by acting as AChEI [15]. While galantamine has been seen to show an initial period of cognitive improvement that lasts 6-12 months, Richarz et al. looked to determine if the efficacy is longstanding beyond this time frame in a prospective open-label trial with 75 patients. The study concluded that galantamine significantly improved cognitive function as ADAS-cog at 12 months showed an improvement from 2.2 to 3 points (P < 0.05). However, after 18-24 months, the ADAS-cog score returned to baseline, and at three years, patients showed an average decline of 2.9 scores, indicating galantamine's efficacy in improving or slowing cognitive impairment [16]. The findings are summarized in Table* *1.

**Table 1:** Role of acetylcholinesterase inhibitors in the management of Alzheimer's disease AD: Alzheimer's disease.

Author, year, country	Study population	Mechanism of action	Benefits	Adverse effects	Contraindications/Cautions
Donepezil: Zhang et al., 2022, China [10]	N = 468	Cholinesterase inhibitor	Improves cognitive and global function in AD patients	Weight loss, diarrhea, nausea, and vomiting	Bradycardia, cardiac conduction disease, urinary incontinence, angina, myocardial infarction, stroke
Rivastigmine: Karaman et al., 2005, Turkey [11]	N= 44	Cholinesterase inhibitor	Improves cognitive function and global clinical function in those with advanced-moderate AD	Nausea, vomiting, dizziness, and abdominal pain	
Galantamine: Wilcock et al., 2001, Europe and Canada [17]	N= 653	Cholinesterase inhibitor	Improved cognitive functions(memory, attention, and language)	Mild to moderate nausea	

NMDA receptor inhibitor

Memantine

Memantine is an FDA-approved medication with an indication for treating symptoms of moderate to severe AD. One of the AD pathophysiology theories proposes that overstimulation of the NMDA receptor by glutamine and subsequent neuronal death is responsible for the cognitive decline seen in AD [18]. Memantine acts as an NMDA receptor antagonist, preventing NMDA overstimulation and neuronal death. A study assessed the reports of memantine delays on cognitive and global function decline using the Clinician’s Interview-Based Impression of Change Plus Caregiver Input (CIBIC-Plus) and Alzheimer's Disease Cooperative Study-Activities of Daily Living Inventory modified for severe dementia (ADCS-ADLsev) [18]. The study's method was to assess the endpoint first, which was the mean differences in treatment at the baseline, and then the goal was to randomly assign a patient a placebo or 20 mg of memantine daily for 28 weeks. The results of the study concluded that patients who received memantine had a better outcome compared to those who received a placebo.

The CIBIC-Plus' assessment at baseline, indicated as “no change,” was set at 4.00, with higher values illustrating the worsening of the condition. Compared to the baseline, the endpoint for the observation of memantine was 4.5 + 1.12 (95% CI: -0.51 to 0.02, P = 0.06), and the last observation carried forward at 28 weeks was 4.4 + 1.13 (95% CI: -0.51 to 0.02, P = 0.06). Compared to the placebo, it was 4.8 + 1.09 (95% CI: -0.51 to 0.02, P = 0.03) at the endpoint and 4.7 + 1.13 (95% CI: -0.51 to 0.02, P = 0.03) after 28 weeks, indicating the changes from baseline [18]. The total ADCS-ADLsev scores indicated that the more negative values from the observation resulted in a worse outcome. The analysis showed -3.1 + 6.79 (95% CI: 0.49 to 3.78, P = 0.02) at the endpoint for memantine and -2.5 + 6.27 (95% CI: 1.45 to 5.28, P = 0.03) at week 28 in the last observation. For the placebo group at the endpoint, it was -5.2 + 6.33 (95% CI: 0.49 to 3.78, P = 0.02), and after 28 weeks, it was -5.9 + 6.78 (95% CI: 1.45 to 5.28, P = 0.003). Therefore, there was significantly less deterioration in the memantine group than in the placebo group [18]. Memantine was well tolerated as most adverse effects were mild to moderate in severity and found to be either statistically insignificant or unlikely to be related to memantine. However, in patients with moderate to severe renal impairment, dosage should be carefully titrated as a study found that exposure to memantine calculated from the area under the plasma concentration-time curve increased by 60% (95% CI: 24%-97%) and 115% (95% CI: 77%-152%), respectively [19]. The findings are summarized in Table 2.

**Table 2:** Role of NMDA inhibitors in the management of Alzheimer's disease USA: United States of America; NMDA: N-methyl-D-aspartate.

Author, year, country	Study population	Mechanism of action	Benefits	Adverse effects
Memantine: Reisberg et al., 2003, USA [18]	N = 252	N-methyl-D-aspartate receptor antagonist	Delays cognitive and global function decline	No significant effects

Monoclonal antibodies

Monoclonal antibody therapies aim to halt and slow the progression of AD by targeting the specific mechanisms of the disease. Specifically, following the amyloid cascade hypothesis, the deposition of beta-amyloid plagues within the brain parenchyma contributes to the physiopathology of AD through neurofibrillary tangles, vascular alterations, microglial, astrocytic activations, and neuronal atrophy and loss. Monoclonal antibodies clear these aggregations of tau or β amyloid plaques and modify the course of the disease. Furthermore, these monoclonal antibodies share similar adverse effects of amyloid-related imaging abnormalities (ARIA), though some may not necessarily include these effects. Amyloid-related imaging abnormalities consist of edema and effusion (ARIA-E) as well as sulcal/leptomeningeal hemosiderin deposits and microhemorrhages (ARIA-H). Currently, FDA-approved monoclonal antibodies for the treatment of AD are aducanumab and lecanemab. Recently proposed monoclonal antibodies under review for advancements in AD include donanemab and semorinemab.

Aducanumab

Aducanumab received accelerated approval in June 2021 with an indication for treating mild AD. Aducanumab is a human monoclonal antibody that targets and removes an aggregated Aβ. A clinical trial demonstrated that high-dose aducanumab significantly delayed cognitive decline, with a difference of -0.39 compared to placebo in Clinical Dementia Rating Sum of Box (CDR-SB) (95% CI: -0.69 to -0.9, P = 0.012). Additionally, at week 78, high-dose aducanumab significantly reduced amyloid positron emission tomography (PET) composite standardized uptake ratio (SUVR) compared to placebo, -0.278 (95% CI: -0.306 to -0.250, P < 0.0001), suggesting a decrease in amyloid plaques. Lastly, the difference in plasma tau protein levels between high-dose aducanumab and placebo was -0.667 (95% CI: -0.860 to -0.474; P < 0.0001), demonstrating aducanumab’s efficacy. About 35% of patients experienced ARIA-E, and 20% of patients with ARIA-E also experienced ARIA-H. The risk of ARIA-H in patients without ARIA-E was not significantly different from that of a placebo. Yet, serious complications of ARIA, such as confusion, delirium, abnormal gait, seizure, and memory impairment, were rarely reported at 1.5%. Other adverse effects included nasopharyngitis, falls, localized superficial siderosis, and dizziness [20]. Given the severe side effects of the medication, regular magnetic resonance imaging (MRI) monitoring is required, and careful clinical assessment of patients is crucial.

Lecanemab

Lecanemab is the most recent AD medication that has received FDA approval in 2023. Lecanemab is a humanized monoclonal antibody that binds and removes Aβ protofibrils. A double-blind study demonstrated that lecanemab slowed the progression of cognition and global function decline compared to placebo groups as the difference in CDR-SB was -0.45 (95% CI: -0.67 to -0.23; P < 0.001). Also, the amyloid burden on PET measured in centiloids was significantly low in lecanemab compared to placebo with a difference of -59.12 centiloids (95% CI: −62.64 to −55.60; P < 0.001) Lecanemab was associated with side effects including infusion-related reactions, ARIA-H, headaches, and falls. Infusion-related reactions were mild to moderate, and preventative medications reduced occurrences for subsequent doses [21]. Further, ARIA was mild to moderate, mostly asymptomatic, and resolved within three months. However, APOE4 carrier patients are at risk of ARIA, and thus APOE genotyping is recommended before starting treatment for better risk discussion with patients [21]. The findings are summarized in Table 3.

**Table 3:** Current monoclonal antibodies for the management of Alzheimer's disease ARIA-E: Amyloid-related imaging abnormalities-edema and effusion; ARIA-H: Amyloid-related imaging abnormalities-hemorrhage; USA: United States of America; PET: Positron emission tomography.

Author, year, country	Study population	Mechanism of action	Benefits	Adverse effects
Aducanumab: Budd Haeberlein et al., 2022, USA [20]	N = 1638	Binds and removes Aβ-soluble oligomers and insoluble fibrils	Decreases plasma tau levels and decreases the amyloid burden on amyloid PET	ARIA-E, ARIA-H nasopharyngitis, localized superficial siderosis, dizziness, confusional state, delirium, gait disturbance, generalized tonic-clonic seizure, and headache
Lecanemab: van Dyck et al., 2023, USA [21]	N = 1795	Binds and removes Aβ-soluble protofibrils	Decreases amyloid burden on amyloid PET, delays progression of cognition and global function decline	Infusion-related reactions, ARIA-H, headaches, and falls

Upcoming innovative treatments: Monoclonal antibody therapies 

Donanemab

Donanemab is a monoclonal antibody that is primarily aimed at clearing insoluble β-amyloid plaques in the brain. Recent studies have found the use of donanemab to be effective in significantly slowing the clinical progression of AD at 76 weeks of treatment, providing clinically meaningful benefits. Researchers of the TRAILBLAZER-ALZ2 trial set the primary outcomes as changes in the integrated Alzheimer’s Disease Rating Scale (iADRS) score from baseline to 76 weeks [22]. Other secondary assessments of cognition for the donanemab treatment group showed slowing clinical progression, decreased brain amyloid plaque levels measured through PET scans, lesser decrease in hippocampal volume, and reduced p-tau217 levels in the treatment population of low/medium baseline tau level participants versus the high tau level participants. The former group appeared to have significant differences in improvements of cognitive scores of iARDS (difference: 3.25 [95% CI: 1.88 to 4.62]; P < 0.001), therefore demonstrating a 35.1% (95% CI: 19.90% to -50.23%) deceleration of disease progression. However, the latter group reported virtually no difference in iARDS outcomes compared to the placebo group (difference: 1.26 [95% CI: -1.77 to 4.28; P = 0.42]) [22]. In the treatment group of donanemab, three participants experiencing serious ARIA-E subsequently expired. These findings may uphold the hypothesis that the treatment of AD at earlier stages is more likely to provide greater clinical benefits at a lower risk of ARIAs. Adverse effects that led to discontinuation of study participation included ARIA-E or ARIA-H, hypersensitivity, and infusion-related reactions [22]. Furthermore, post hoc analysis studies of donanemab noted a correlation at 24 weeks of amyloid reduction due to donanemab treatments and the study participant’s baseline amyloid levels, which slowed the accumulation of tau (spearman correlation coefficient r: -0.54; 95% CI: [-0.66 to -0.39], P < 0.001) [23]. These associations corroborate the findings that amyloid clearance levels influenced the amount of tau accumulation inhibition.

Semorinemab

Semorinemab is an antitau monoclonal antibody. Recent phase 2 clinical trials suggested that semorinemab demonstrated no clinical difference compared to placebo trials in clinical outcomes of cognitive evaluations and clinical decline from baseline when utilizing the CDR-SB score (placebo: difference 2.19 [95% CI: 1.74 to 2.63]; semorinemab 1500 mg: difference 2.36 [95% CI: 1.83 to 2.89]; semorinemab 4500 mg: difference 2.36 [95% CI: 1.92 to 2.79]; and semorinemab 8100 mg: difference 2.41 [95% CI, 1.88 to 2.94]). However, researchers noted that additional studies may be needed to evaluate the clinical utility of antitau monoclonal antibodies in the future. Additionally, it seemed that although there may not have been a statistical clinical benefit, cerebrospinal fluid (CSF) tTau and pTau181 analysis of variance seemed to show reductions relative to placebo group levels from baseline but were limited by small sample sizes (CSF tTau; week 49: F3,59 = 3.73, P = 0.02; week 73: F3,46 = 2.80, P = 0.05 and pTau181; week 49: F3,59 = 4.05, P = 0.01; week 73: F3,46 = 3.79, P = 0.02). Additional pooled data of semorinemab treatments demonstrated more significant reductions compared to placebo for CSF tTau (week 49; t61 = 3.23, P = 0.002; week 73: t48 = 2.19, P = 0.03) and pTau181 (week 49: t61 = 3.21, P = 0.002; week 73: t48 = 3.07, P = 0.004) [24]. Adverse effects were mild and uncommon. These included events such as falls, infections, nasopharyngitis, infusion reactions, and ARIAs, which resolved spontaneously. Similarly, the Phase II Lauriet study noted that the treatment participants in the semorinemab treatment group had no statistically significant difference from baseline versus placebo in terms of assessing scores of the Alzheimer’s Disease Cooperative Study-Activities of Daily Living Scale (ADCS-ADL) (difference: -0.83; 95% CI: -3.39 to 1.72; P = 0.52). However, at week 49, there was a 42.2% reduction in the decline of scores on the ADAS-cog11 neurocognitive assessment compared to the placebo (difference: -2.89; 95% CI: -4.56 to -1.21; P = 0.0008) [25]. The findings are summarized in Table 4.

**Table 4:** Monoclonal antibody treatments for Alzheimer's disease AD: Alzheimer's disease; ARIA: Amyloid-related imaging abnormalities.

Author, year, country	Study population	Mechanism of action	Benefits	Adverse effects
Donanemab: Sims et al., 2023, United States [22]	N = 1736	β-amyloid epitope monoclonal antibody	Significant amyloid plaque reduction slows clinical Alzheimer’s cognitive progression, improves integrated Alzheimer’s Disease Rating Scale scores, lowers the decrease in hippocampal volume, and reduces p-tau217 levels.	Amyloid-related imaging abnormalities-edema, amyloid-related imaging abnormalities-effusion, infusion-related reactions, microhemorrhages, hemosiderin deposits, and hypersensitivity
Semorinemab: Teng et al., 2022, USA [24]	N = 457	Antitau antibody	No clinical efficacy in slowing AD clinical decline. May have reduced CSFTau and pTau181 levels slightly.	Mild effects of infusion reactions as well as spontaneously resolving ARIAs

Note that other studied monoclonal antibody phase 3 trials such as gantenerumab, solanezumab, bapineuzumab, and crenezumab were negative, which showed futility in halting the progression of AD, even exacerbated the patient’s symptoms, or showed harmful adverse effects; therefore they were not included in this review of upcoming AD treatments.

Tau and amyloid-targeting treatments

Methylthioninium Moiety

Methylthioninium moiety (MT) has been previously studied in Phase II trials as monotherapy for the treatment of AD due to its function in tau protein aggregation inhibitor activity [26]. Extrapolating upon this, leuco-methylthioninium bis (hydromethanesulphonate) or LMTM is an oxidized form of this methylthioninium moiety that is being developed as a treatment for AD based on inhibition of tau aggregation. LMTM was studied as a monotherapy in an 18-month Phase III clinical trial in a non-randomized cohort of patients with AD [27]. The brain atrophy rate in patients on the monotherapy compared to normal elderly controls demonstrated a significant decline in brain atrophy after nine months of LMTM monotherapy (difference: 4.7 [95% CI: 1.0 to 8.5; P = 0.0068]).

Tramiprosate

Tramiprosate works by inhibiting the Aβ monomer aggregation into toxic oligomers and is studied for the implications of cognitive Alzheimer’s symptoms. Two double-blind, placebo-controlled studies were conducted to determine the efficacy and safety of the treatment and the appropriate stage for treatment initiation. One study showed that the lower dose arm had higher ADAS-cog scores versus placebo and higher dose arms but a lower CDR-SB baseline [28]. On the other hand, one European Union study revealed that the placebo group had higher ADAS-cog and CDR-SB scores compared to the active dose arms. However, in retrospective analyses, researchers determined that the 160 mg BID tramiprosate treatment reached the highest efficacy rates for least square mean differences in APOE4/4 carriers with mild AD patients for scores in ADAS-cog, CDR-SB, and Disability Assessment for Dementia (DAD) evaluations in comparison to the overall mild Alzheimer’s group (-4.8, P = 0.001; -0.9, P = 0.05; 8.4, P = 0.02). These cognitive effects stabilized over the 78 weeks and were found to have no decline and increased over time [28]. Those with higher MMSE 22 baseline scores of 22 and above had greater beneficial effects on the tramiprosate treatment compared to the generally mild and moderate AD group. As opposed to monoclonal antibody treatments, tramiprosate did not have any evidence of vasogenic edema/ARIA-edema. Adverse effects were mild to moderate and included nausea, vomiting, depression, and decreased weight. These results can be utilized to focus on future studies of genetic-specific APOE4 homozygote Alzheimer’s patients [28]. The findings are summarized in Table 5.

**Table 5:** Drugs targeting tau and amyloid USA: United States of America; LMTM: Leuco-methylthioninium bis (hydromethanesulphonate); MMSE: Mini-mental State Exam; ADAS-cog: Alzheimer’s Disease Assessment Scale-Cognitive Subscale; DAD: Disability Assessment for Dementia; ARIA-E: Amyloid-related imaging abnormalities-edema and effusion.

Author, year, country	Study population	Mechanism of action	Benefits	Adverse effects
LMTM: Wischik et al., 2015, Scotland [26]	N = 321	Tau aggregation inhibitor	Preventing clinical decline	Gastrointestinal (primarily diarrhea), renal and urinary disorders, and falls
LMTM: Wilcock et al., 2018, Scotland [27]	N = 800	Tau aggregation inhibitor	Whole brain and lateral ventricular volume	Urinary tract disorders, gastrointestinal disorders (nausea and vomiting), and depression
Tramiprosate: Abushakra et al., 2017, USA [28]	N = 2025	Binds soluble amyloid β, inhibiting aggregation	Improved MMSE 20-26 scores, ADAS-cog, and DAD cognition scores, no evidence of vasogenic edema/ARIA-E	Nausea, vomiting, depression, and decreased weight

Anti-neuroinflammation, antidiabetic, and miscellaneous agents

NE3107

NE3107 is an oral insulin sensitizer that addresses the role of insulin sensitization in neurodegeneration. It has been shown to bind extracellular signal-regulated kinase and inhibit inflammation-driven extracellular signal-regulated kinase (ERK) and nuclear factor kappa-light-chain-enhancer (NFkB)-stimulated inflammatory mediators. A Phase III study is currently being done by Bio Vie, which will be completed in November 2023 [29].

Metformin

Metformin is a first-line drug in individuals with diabetes to help prevent hyperglycemia and promote weight loss. In observational studies, metformin has been observed to have a reduced incidence of dementia in diabetic patients. A recent study in 2022 by Zheng et al. looked at a database of patients for metformin-targeting proxies and their associated cognitive outcomes [30]. Results indicated that genetically proxied metformin use resulting in a reduction of 6.75 mmol/mol (1.09%) in HbA1c levels was associated with 4% lower odds of AD risk and increased cognitive function (OR 0.96 [CI 95%: 0.95, 0.98]; P = 0.000106). Therefore, it was concluded that metformin use may prevent AD risk via various mechanisms.

Sodium Oligomannate

Sodium oligomannate (GV-971) is an oligosaccharide that inhibits the neurotoxicity of Aβ aggregation by inhibiting accumulation and decreasing polymerization of the Aβ, and this medication was approved in China but not cleared by FDA yet. This decreases the accumulation of toxic fibrils into non-toxic monomers and also decreases neuroinflammation. A recent study conducted by the Shanghai Institute of Materia Medica demonstrated that there was a significant and sustained improvement over 36 weeks in cognition in patients who had administration of GV-971 as compared to those with placebo administration (difference: -2.15 [CI 95%: -3.07 to -1.23; P < 0.0001; effect size: 0.531]) [31].

Immunoglobulin

Trials of intravascular immunoglobulin (IVIG) infusions demonstrate statistically significant decreases in plasma A β42 levels, a major constituent of plaques (0.4g/kg treatment: P = 0.001, 0.2 g/kg treatment: P = 0.001) [32]. However, there were no changes in the plasma Aβ-40 levels. These findings may be due to the selective binding of self-assembling Aβ42 molecules by antibodies that target specific conformations of amyloid. The changes between treatment and placebo for cognitive test scores of ADAS-cog and ADCS-ADL over the 18-month treatment course were not statistically significant. Subsequent neurological test scores also did not show significant differences between the treatment and placebo control groups. Adverse effects from the treatment included rashes from infusion reactions, decreases in hemoglobin levels, chills, arthralgia, vomiting, epistaxis, occasionally eczema, and upper respiratory tract infections [32].

Masitinib

Masitinib is a tyrosine kinase inhibitor that impacts mast cells and microglia of the neurological system. Though not a direct treatment, study AB09004 analyzed the impacts of masitinib as an adjunct treatment to more established cholinesterase inhibitor treatments and memantine in those with mild to moderate AD. Results concluded that the treatment group of 4.5 mg/kg/day masitinib improved significantly over the placebo group solely on the primary outcome of ADAS-cog test scores at the end of 24 weeks (difference: -2.15; 97.5% CI: [-3.48, -0.81]; P < 0.001) [33]. The parallel group of 6.0 mg/kg/day masitinib dose, however, did not show any significant differences from the placebo group on cognitive function tests of ADAS-cog or ADCS-ADL (difference: −0.43; 97.5% CI: [−1.81, 0.95], P = 0.483) [33]. The 6.0 mg/kg/day treatment arm improved from baseline ADCS-ADL scores but seemed to have an atypical improvement [33]. For now, the most beneficial dose of masitinib in clinical development seems to be 4.5 mg/kg/day. Adverse effects of masitinib were mild or moderate and included neutropenia, Stevens-Johnson syndrome, which was non-life threatening and inconclusive, and pneumonia. Adjunctive treatments of masitinib in addition to a cholinesterase inhibitor and/or memantine would be a viable treatment option in the future to benefit mild to moderate Alzheimer's patients [33]. The findings are summarized in Table 6.

**Table 6:** Anti-inflammation, antidiabetic, and miscellaneous agents to manage Alzheimer's disease UK: United Kingdom; MR: Mendelian randomization; MCI: Mitochondrial complex 1; NDUFA2: MCI-related gene; AD: Alzheimer's disease.

Author, year, country	Study population	Mechanism of action	Benefits	Adverse effects
Metformin: Zheng et al., 2022, UK [30]	N = 24,087	Antidiabetic agent, increases insulin sensitivity, MR analysis of molecular phenotypes	Independent role of inhibition of MCI on reducing Alzheimer’s disease risk. NDUFA2, a mitochondrial-related gene, is identified as a key mediator in the brain.	None noted
Sodium oligomannate: Xiao et al., 2021, China [31]	N = 818	Anti-neuroinflammatory	Improved cognition with sustained improvement in AD patients	Hyperlipidemia, nasopharyngitis, upper respiratory infection, elevated blood glucose, dizziness, and hyperlipidemia
Masitinib: Dubois et al., 2023, France, Spain, South Africa, Greece [33]	N = 718	Tyrosine kinase inhibitor targeting activated mast cells and microglia	Significant improvements in cognitive function based on the Alzheimer’s Disease Assessment Scale-Cognitive Subscale	Neutropenia, pneumonia, and Steven-Johnson syndrome

Phase 2 trials of anti-inflammatory agents

Atomoxetine

Atomoxetine is a selective norepinephrine reuptake inhibitor proposed for neuroprotection use in mild AD. Phase 2 clinical trials of atomoxetine substantiate the potential for its use in clinically improving AD. Researchers obtained baseline, six-month, and 12-month evaluations of neuropsychological testing; collected CSF amyloid-β42, tau, and pTau181 levels; and obtained brain MRI and fluorodeoxyglucose-positron emission tomography (FDG-PET) results of participants [34]. For the most part, all but two participants completed the treatment trials to the maximum titrated dose of 100 mg of atomoxetine. Side effects were also similar between placebo and treatment groups, among those being dizziness, dysautonomia, gastrointestinal symptoms, and dry mouth. As the trial was only conducted for six months, researchers expected the result of no statistical difference in cognition function ADAS-cog evaluations (P = 0.31) [34]. Resting-state functional MRI (fMRI) scan of atomoxetine treatment patients seemed to show significantly increased inter-network connectivity in the regions of the insula and hippocampus (P = 0.05) but decreased inter-network connectivity between the inferior frontal gyrus and caudate networks [34]. Additionally, FDG-PET scans of the treatment group also demonstrated a significant increase in glucose uptake in the hippocampus (P = 0.036), parahippocampal gyrus (P = 0.023), middle temporal pole (P = 0.021), inferior temporal gyrus (P = 0.022), and fusiform gyrus (0.027) regions [34]. CSF levels of pTau181 and total tau reduced slightly with treatment, and other brain metabolism proteins such as astrocyte and microglia-linked neuroinflammatory processes seemed to normalize. Overall, atomoxetine is well tolerated with mild side effects and modifies the pathophysiology of biological markers of AD and neuroinflammation even if there are no clinical effects. These disease-modifying properties warrant further investigation [34].

Losartan

Losartan acts antagonistically against the angiotensin 2 receptor. Utilizing this modification of the angiotensin 2 signaling, researchers investigate its use of reducing Alzheimer’s pathology and brain volume loss for mild to moderate disease progression. A Phase II trial has measured changes in whole brain volume between baseline and the 12 months of losartan treatment in which researchers found that there was no statistical effectiveness in reducing brain atrophy in mild to moderate Alzheimer’s patients. Specifically, there were no statistical differences in the changes to hippocampal atrophy and ventricular volume (difference: -2.29 mL; 95% CI: [-6.46 to 0.89]; P = 0.14) [35]. Despite no changes in brain volume, it was noted in the NILVAD sub-study that losartan increased hippocampal blood flow. The losartan treatment group experienced greater reductions in systolic and diastolic blood pressures. Adverse effects were mild and mostly included infections, mechanical injury, neuropsychiatric, and gastrointestinal illnesses. While patients tolerated losartan well, researchers theorized that it may not have crossed the blood-brain barrier to enact brain effects. Additional studies investigating the effects of losartan on patients with earlier Alzheimer’s progression and longer treatment periods may be needed to fully understand the potential of losartan’s use in halting AD progression [35].

Genistein

Genistein has also been recently studied as an isoflavone neuroprotective therapeutic for prodromal AD in the hopes of lowering brain inflammation. In the GENIAL study, there was no statistical difference between genistein and placebo for primary outcomes of improving with flutemetamol PET in different regions of the brain at six months (P = 0.878). On the other hand, there seemed to be a statistically significant improvement in the genistein treatment groups in the dichotomized direct Complutense Verbal Learning Test (TAVEC) (p=0.031) and the dichotomized delayed Centil REY Figure Test copy (P = 0.002) [36]. It was noted that in the anterior cingulate gyrus area, genistein-treated patients accumulated fewer amyloid deposits at 12 months of treatment. Secondary cognitive outcomes also indicated, albeit with exploratory results, that the genistein-treated patients achieved significant improvements in cognitive tests. No severe adverse effects were reported in the study, with the main side effect simply being mild diarrhea. Further studies may be needed to validate these promising results with more patients and for a longer time.

Trans-resveratrol studies have additionally demonstrated its potential for neuroprotective roles in the treatment of moderate to mild AD as a polyphenol antioxidant plant estrogen that affects estrogen receptors. Specifically, on cognitive tests such as the MMSE and ADCS-ADL, patients treated with trans-resveratrol showed a greater slowed decline in scores in comparison to the placebo group (P = 0.06) [37]. Alongside these cognitive improvements, it was also found that patients treated with trans-resveratrol had a decrease of CSF matrix metallopeptidase nine levels from baseline to week 52, potentially indicating a reduction in inflammation pathology. The placebo group showed no such reductions in CSF matrix metallopeptidase levels. It should be noted that brain volume seemed to decline more in the treatment group (P = 0.033); however, the decline did not seem to correlate with cognitive decline [37]. The main adverse effects included headache, diarrhea, nausea, back pain, weight loss, and rash. Continued future research on trans-resveratrol should include larger studies including more patients with mild to moderate AD to validate these findings and investigate other markers of inflammation for brain cell death [37].

Benfotiamine

Benfotiamine is a synthetic thiamine precursor that affects many enzymes and direct actions on inflammation and oxidative stress. Benfotiamine activates transketolase that accelerates the decrease of the precursors of advanced glycation end-products, which contribute to Alzheimer’s plaque formation. It also decreases metabolic stress, which decreases vascular complications. Furthermore, there is an increase in blood thiamine concentration, and benfotiamine may overcome the reduction in activity of thiamine-dependent diphosphate enzymes. In a study by Gibson et al., 2020, there appeared to be an improvement in 36 patients (as compared to 34 placebo) in cognitive function and decreased levels of advanced glycation end-products (P = 0.044) with no adverse effects reported in the treatment group. These protective effects were especially effective in the APOE ɛ4 noncarriers. The benfotiamine group had a 43% lower increase in ADAS-cog scores (P = 0.125) than the placebo group, indicating nearly statistically significant less cognitive decline [38].

Other drugs previously investigated or in progress

Beta-Site APP Cleaving Enzyme-1 Inhibitor

The beta-site APP cleaving enzyme-1 (BACE1) inhibitor has been investigated for its potential use in reducing the clinical decline of AD; however, studies have shown little efficacy. One Phase III EPOCH trial showed no evidence of improved cognitive benefits after 78 weeks of treatment with the BACE inhibitor, verubecestat. However, it should be noted that during the treatment, MRI assessments showed accelerated volumetric loss within the brain regions of the hippocampus and other amyloid-predominant brain areas (placebo P = 0.961; 12 mg P = 0.443; 40 mg P = 0.021) [39].

Vafidemstat

Trials have also been in progress for the histone lysine-specific demethylase KDM1A, vafidemstat. Rat models were genetically modified to simulate advanced aging, and AD showed improvements in memory rescue with the administration of vafidemstat in conjunction with the selective monoamine oxidase type B (MAO-B) inhibitor rasagiline. Additional benefits included reduced aggression and ameliorated social behavior. Results of Phase 2a trials in human populations have also been presented at conferences; however, there have not yet been any written publications surrounding the ETHERAL-US trials of the drug ORY-2001 (vafidemstat). Conference data demonstrated the MAO-B inhibitor to be a safe and well-tolerated drug; however, there did not seem to be cognitive improvements after six months of drug administration. A longer clinical trial may be needed to assess the efficacy and trials of the drug properly, and the trials of the drug are still ongoing [40].

Combined Metabolic Activators

Combined metabolic activators (CMA) have also been assessed for AD cognitive benefits. A Phase II study assessed cognitive function and cognitive assessment score improvements (P = 0.0073) along with hippocampal brain volumes and cortical thickness while undergoing treatment with CMA. Results of the study concluded that administration of CMA seemed to improve cognitive functions and cognitive score assessments, and brought about statistically significant alterations to protein plasma levels and metabolites in nicotinamide adenine dinucleotide (NAD+) and glutathione metabolism, which indicated the efficacy of CMA [41].

Efavirenz

Other drugs for AD potential include the anti-HIV antiviral efavirenz, which activates cytochrome P450 46A1 (CYP46A1) to process cholesterol in the central nervous system (CNS) into 24-hydroxycholesterol (24HC). Phase I trial EPAD (efavirenz for patients with Alzheimer’s disease) recruited participants with early AD to evaluate whether low-dose efavirenz would induce CYP46A1 activity. Ultimately, the study found statistically significant improvements in cholesterol turnover into 24HC from baseline (P ≤ 0.001), with no serious adverse effects. Further trials should be conducted to determine cognitive benefits [42].

Bromocriptine

Bromocriptine, a dopamine D2 agonist, may be useful in the case of patients with presenilin 1 (PSEN1) mutations that lead to early onset AD. Researchers caution against the use of bromocriptine in combination with CYP3A4 inhibitors/inducers and dopamine antagonists since they might result in increased adverse effects. Trials and publications are underway to determine the safety and efficacy of bromocriptine in AD. Prior safety and efficacy of bromocriptine have already been shown in the use of Parkinson’s disease [43].

AD-associated symptom treatments

Brexpiprazole

Brexpiprazole acts as a serotonin dopamine activity modulator that is a partial agonist for the serotonin 5-HT1A and dopamine D2 receptors. It also has antagonistic activity at the serotonin 5-HT2A receptor and noradrenaline α 1B/α 2C. Typically used to treat schizophrenia as an antipsychotic, brexpiprazole is also being investigated as a treatment for the outward symptoms of AD agitation. Research has found that treatment of brexpiprazole at higher fixed doses of 2 mg improved Cohen-Mansfield Agitation Inventory Scores (adjusted mean difference: −3.77; confidence limits: -7.38, -0.17; t(316) = -2.06; P = 0.040; MMRM). Despite this, brexpiprazole 1 mg/day did not show statistically significant improvements (0.23; −3.40, 3.86; t(314) = 0.12; P = 0.90; MMRM) [44]. Some patients experienced mild or moderate symptoms of seizure, headache, somnolence, insomnia, dizziness, urinary tract infection, and chronic obstructive pulmonary disease; otherwise, the treatment was well tolerated with little to no side effects in most.

Suvorexant

Suvorexant is an orexin receptor antagonist studied for the treatment of insomnia in mild to moderate AD patients. About 10 mg of suvorexant was compared to placebo groups to measure the primary outcomes of change from baseline in total sleep time measured by polysomnography at the end of four weeks; the results demonstrated least squares mean improvement from baseline by 73 minutes and only 45 minutes for placebo (difference: 28 minutes; CI 95%: 11‐45; P < 0.01). The results showed improved total sleep time in patients with increased rapid eye movement (REM) sleep. The suvorexant treatment also seemed to reduce the time taken to reach REM sleep. Patients experienced some adverse effects of somnolence, headache, dry mouth, and hypnagogic/hypnopompic hallucinations [45].

Supplements enhancing cognition and slowing AD decline

Melatonin

Melatonin is a hormone that is released by the pineal gland that regulates the circadian rhythm of the body. As a supplement, it also functions as a neuroprotective molecule against the pathogenesis of AD. Multiple studies have shown that melatonin can diminish Aβ generation by enhancing non-amyloidogenic APP processing and inhibiting Aβ assembly. Melatonin will modulate the levels of various membrane and signaling proteins such as ADAM10, BACE1, PIN1, and GSK3. As a result, it reduces Aβ production. Melatonin can exert its neuroprotective role by enhancing Aβ clearance through pathways that include glymphatic and lymphatic drainage, BBB transportation, and autophagy, thereby preventing the buildup and development of plaques in the brain. Many studies have shown that melatonin could attenuate Aβ oligomer-induced neurotoxicity by modulating various mechanisms. Some of these mechanisms include the PrPc/mGluR5/Fyn/Pyk2, Ca2+/mitochondria, and miR-132/PTEN/Akt/FOXO3 pathways. Lastly, melatonin has shown improvements in cognitive function and sleep quality in patients with AD. By maintaining continuous melatonin supplementation, melatonin has the underlying benefit of reducing the risk of the neurodegenerative process of Aβ accumulation in elderly individuals [46].

Eicosapentaenoic Acid

Eicosapentaenoic acid (EPA) is an omega-3 fatty acid, which is a nootropic agent studied for anti-inflammation and cognitive preservation. It is also studied to be beneficial for brain development. Research has shown that EPA could significantly decrease lipid peroxidation levels and prevent apoptosis. In addition, EPA inhibits hyperphosphorylation of tau suggesting that it could improve cognitive impairment in Aβ1-42-induced AD. A study was conducted in participants aged 84 ± 3 years in a multicenter cohort study. The results showed that higher concentrations of EPA were associated with a lower incidence of AD in accordance with a hazard ratio of .76 in a follow-up window of seven years, indicating the beneficial role of EPA for cognitive health in old age (95% CI: 0.63, 0.93; P = 0.008) [47].

Alpha-Tocopherol

Alpha-tocopherol (vitamin E) is a lipid-soluble vitamin that acts as an antioxidant molecule by removing free radicals’ toxic effects on cells. In animal models, it was demonstrated that vitamin E supplementation reduced androgen-binding protein-induced cell death [48]. In addition, a study measured the time taken by AD patients to deteriorate to one of the following conditions: death, institutionalization, loss of the ability to perform basic activities of living, or severe dementia defined by Clinical Dementia Rating. The study found that patients who took vitamin E supplementation had a median of 670 days (P = 0.001) until deteriorating to one of the above-mentioned conditions, whereas the placebo group had a median of 440 days [49]. Vitamin E supplementation is generally well tolerated by patients. Notable adverse events observed during the above-mentioned clinical trials were dental problems requiring treatment, falls, and syncopal episodes, but such adverse events were not replicated in another clinical trial [49,50].

Caprylidene

Caprylidene is a medical food containing excessive amounts of caprylic acid. As caprylic acid is metabolized to ketone bodies to be utilized by neurons, it is thought to compensate for the decrease in glucose utilization commonly seen in AD patients, especially with the ApoE4 allele [51]. A study reported that AD patients who received caprylidene had significantly lower cognitive impairment reflected by ADAS-cog score differences. In the intention-to-treat population, patients who received caprylidene had a 4.77-point difference (P = 0.0005) from the baseline on day 45 and a 3.36-point difference (P = 0.0148) on day 90 compared to placebo. In the per-protocol population, patients who received caprylidene had a 5.73-point difference (P = 0.0027) on day 45 and a 4.39-point difference (P = 0.0143) on day 90. Finally, in the dosage-compliant population, patients who received caprylidene had a 6.26-point difference (P = 0.0011) on day 45 and a 5.33-point difference (P = 0.008) on day 90. Although caprylidene is well tolerated, side effects include diarrhea, bloating, and indigestion due to its high triglyceride content [52]. Another study done in Japan reported that gastrointestinal side effects were lower in patients who do not typically consume a high-fat diet, indicating that adverse effects of caprylidene could be reduced with a decrease in fat consumption [51].

## Conclusions

AD remains a significant public challenge due to its progressive nature and lack of definite treatments that stop or reverse its progression. Combining one or more of these most recently tested drugs with one or more of the traditional pharmacological agents could become a future viable treatment option for AD. As of now, the combination of monoclonal antibodies and anticholinesterases has not been a standard treatment approach, but research in AD is ongoing. Current mainstay treatment focuses on symptomatic relief. However, recent advancements in monoclonal antibody therapies such as aducanumab and lecanemab have proven effective in lowering Aβ plaques and slowing down cognitive decline. Many innovative therapies are still in the experimental phase such as atomoxetine (selective norepinephrine reuptake inhibitor), losartan (angiotensin 2 receptor agonist), genistein (anti-inflammatory isoflavone neuroprotective agent), trans-resveratrol (polyphenol antioxidant plant estrogen), and drugs targeting AD-associated symptoms such as brexpiprazole (serotonin dopamine activity modulator) and suvorexant (orexin receptor antagonist), with more investigation and clinical research studies required for full approval. While these novel treatments in clinical trials offer hope, many challenges remain, including the need for further research to refine their efficacy, safety profiles, and optimal dosage. Ongoing clinical trials and research efforts continue to pave the way for a deeper understanding of AD's pathophysiology and the development of more effective and targeted therapies. Collaborative efforts among researchers, clinicians, and pharmaceutical companies are essential to bridge the gap between scientific discoveries and transformative therapies, providing relief to individuals and families affected by AD.
